# Linked color imaging improves the diagnostic accuracy of eosinophilic esophagitis

**DOI:** 10.1002/deo2.146

**Published:** 2022-07-25

**Authors:** Yasuhiko Abe, Yu Sasaki, Makoto Yagi, Naoko Mizumoto, Yusuke Onozato, Takashi Kon, Masakuni Shoji, Kazuhiro Sakuta, Takayuki Sakai, Matsuki Umehara, Minami Ito, Shuhei Nakamura, Hidemoto Tsuchida, Yoshiyuki Ueno

**Affiliations:** ^1^ Division of Endoscopy Yamagata University Hospital Yamagata Japan; ^2^ Department of Gastroenterology Faculty of Medicine Yamagata University Yamagata Japan

**Keywords:** endoscopic diagnosis, eosinophilic esophagitis, inter‐observer agreement, intra‐observer agreement, linked color imaging

## Abstract

**Objectives:**

To assess the usefulness of linked color imaging (LCI), a recently developed image‐enhanced endoscopy technique, in the endoscopic diagnosis of eosinophilic esophagitis (EoE).

**Methods:**

Thirty white light images (WLIs) and 30 WLI+LCI images collected from patients with and without EoE were randomly and blindly reviewed by 10 endoscopists, including four experts (Exs) and six non‐Exs. Edema, ring, exudate furrows, and strictures were rated on the adjusted EoE endoscopic reference score; the diagnosis of EoE was assessed. Using the kappa value, inter‐ and intra‐observer agreements were analyzed among endoscopists.

**Results:**

WLI+LCI images had a higher diagnostic accuracy for EoE than WLIs (0.85 vs. 0.70, respectively), especially in non‐Exs or endoscopists with no experience with EoE patients. Inter‐observer agreement for WLI+LCI images statistically surpassed WLIs for furrows (kappa, 0.73 vs. 0.67, respectively; *p* = 0.0013), stricture (kappa, 0.51 vs. 0.39, respectively; *p* = 0.0072), and diagnosis (kappa, 0.67 vs. 0.57, respectively; *p* < 0.0001) of EoE. The increase in inter‐observer agreement in WLI+LCI images allowed for a reduction in the differences between the Exs and non‐Ex endoscopists. Intra‐observer agreement for WLI+LCI images surpassed WLIs for a ring (kappa, 0.62 vs. 0.43, *p* = 0.0052), and a similar trend was found in exudates, furrows, and diagnosis irrespective of the Exs or non‐Exs.

**Conclusions:**

LCI can contribute to the improvement of the endoscopic diagnosis for EoE, with “moderate” to “substantial” consistency, by enhancing the visibility of abnormal findings, leading to reduced diagnostic disparities among endoscopists.

## INTRODUCTION

Eosinophilic esophagitis (EoE) is a chronic immune‐mediated inflammatory disease characterized by esophageal symptoms and intense eosinophilic infiltration localized to the esophagus.^1^ EoE has recently been identified as a major cause of dysphagia and food impaction in adolescents and adults.^2^, ^3^ In EoE, several characteristic endoscopic findings, such as rings, stricture, linear furrows, white exudates, and edema have been reported.^4^ More intense eosinophil infiltration has been demonstrated in exudates, furrows, or the mid‐ to lower esophagus, albeit with a heterogeneity of eosinophil distribution.^5^, ^6^, ^7^, ^8^ Symptoms with the endoscopic findings suggesting EoE, greatly increase the likelihood of a histologically definitive diagnosis of EoE.^9^, ^10^ Thus, endoscopy and appropriate biopsy are vital for the diagnosis of EoE.

Meanwhile, the diagnostic ability of the abovementioned endoscopic findings in EoE has been shown to be unsatisfactory by a previous meta‐analysis, with a lower sensitivity of 15%–48% and lower positive predictive value of 51%–73% against a desirable higher specificity of 90%–95% and higher negative predictive value of 74%–84%.^4^ Therefore, multiple esophageal biopsies are still inevitable when EoE is clinically suspected.^11^ To assess endoscopic findings objectively, the EoE endoscopic reference score (EREFS) has been recently developed, in which edema, rings, exudates, furrows, and stricture are assessed together.^12^ The system improves diagnostic accuracy and is used to evaluate treatment responsiveness,^13^, ^14^ whereas the respective endoscopic finding is noted to be inconsistent.^15^, ^16^, ^17^

Linked color imaging (LCI) is a newly developed image‐enhanced endoscopy (IEE) created by short‐wavelength narrow‐band laser light combined with white laser light, enabling brighter light in a distant area and enhancing color differences between red and white.^18^, ^19^ Accumulating evidence has shown that LCI is helpful not only for the diagnosis of pre‐cancerous/cancerous lesions in the GI tract^20^, ^21^, ^22^, ^23^, ^24^, ^25^, ^26^ but also for some inflammatory conditions such as reflux esophagitis,^27^, ^28^ *Helicobacter pylori* gastritis,^29^ and ulcerative colitis.^30^ However, the usefulness of this technology for the endoscopic diagnosis of EoE remains unclear.

Given the current situation where several endoscopists use both white light image (WLI) and IEE in their daily practice, we investigated whether endoscopic observation using additional LCI improves the accuracy of endoscopic diagnosis of EoE compared to WLI alone.

## METHODS

### Study design

This is a preliminary retrospective study to investigate whether LCI, in addition to WLI, contributes to the improvement of endoscopic diagnosis of EoE. We compared the diagnostic accuracies for EoE using WLI images only and using combined corresponding WLI and LCI images (WLI+LCI) collected from patients with and without EoE. This study was approved by the Ethical Review Committee of Yamagata University Faculty of Medicine (2019‐32). The protocol of this study was disclosed on the web page of our institution, and patients were allowed to refuse to participate in this study by opt‐out consent.

### Preparation of endoscopic images

**FIGURE 1:**
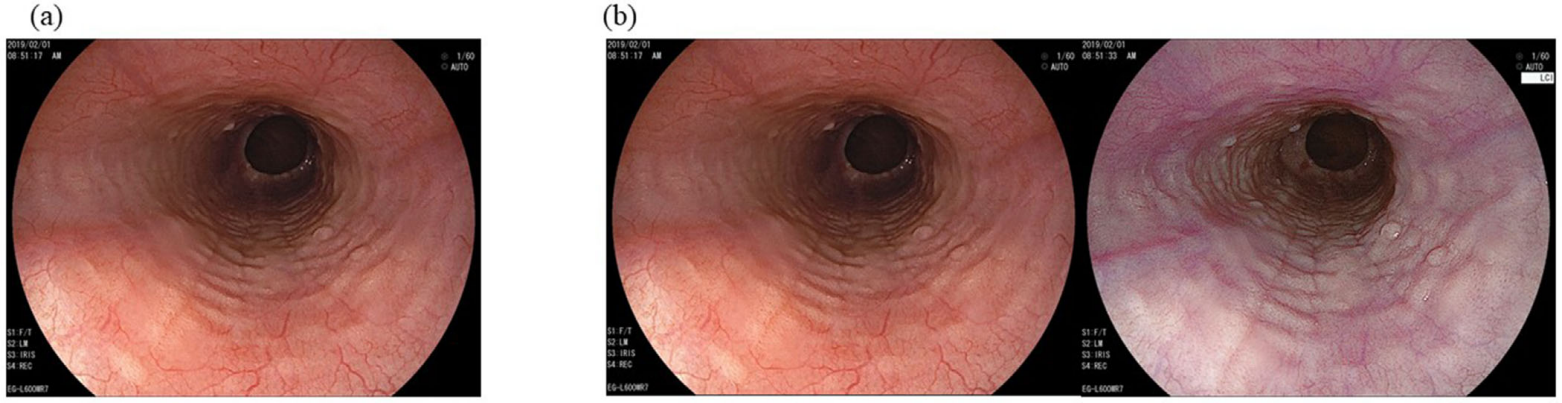
Representative images with the diffuse type of eosinophilic esophagitis in this study. (a) White light image. (b) White light image (left) combined with a linked color image (right)

**FIGURE 2:**
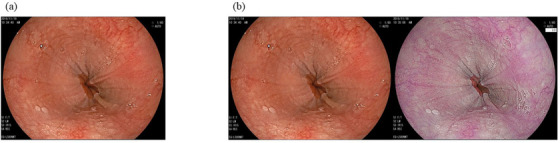
Representative images of a localized type of eosinophilic esophagitis in patients of this study. (a) White light image. (b) White light image (left) combined with a linked color image (right)

Thirty WLI images and 30 WLI+LCI images were collected from consecutive 19 EoE and 30 non‐EoE patients diagnosed at our hospital between March 2018 and April 2021. In some of the 19 EoE patients, two or three different image sets were redundantly extracted. EoE was histologically proven to have eosinophilic inflammation, with a peak of more than 15 eosinophils/high‐power field by obtaining more than two biopsy samples. EoE was classified into two subtypes: diffuse type and localized type according to the endoscopic phenotype with histological supporting findings as previously reported^31^ Thus, the diffuse type was defined as a widespread area of eosinophilic inflammation involving one or more of three locations: upper, middle, and lower esophagus. Multiple biopsies were obtained from the esophagus for endoscopically suspected diffuse‐type EoE. The localized type was defined as a small area of eosinophilic inflammation localized within 1–2 cm of the lower esophagus. In the localized type, at least one biopsy demonstrating eosinophil infiltration ≤5 eosinophils/high‐power field was sampled from the mucosa with a normal appearance above the affected area. Of the 19 EoE patients, 15 had the diffuse type (Figure 1a,b), and the remaining four had the localized type (Figure 2a,b). Finally, 30 WLI and 30 WLI+LCI images were extracted from 19 EoE patients: 25 WLI and 25 WLI+LCI images from 15 diffuse EoE patients and five WLI and five WLI+LCI images from four localized EoE patients.

**FIGURE 3:**
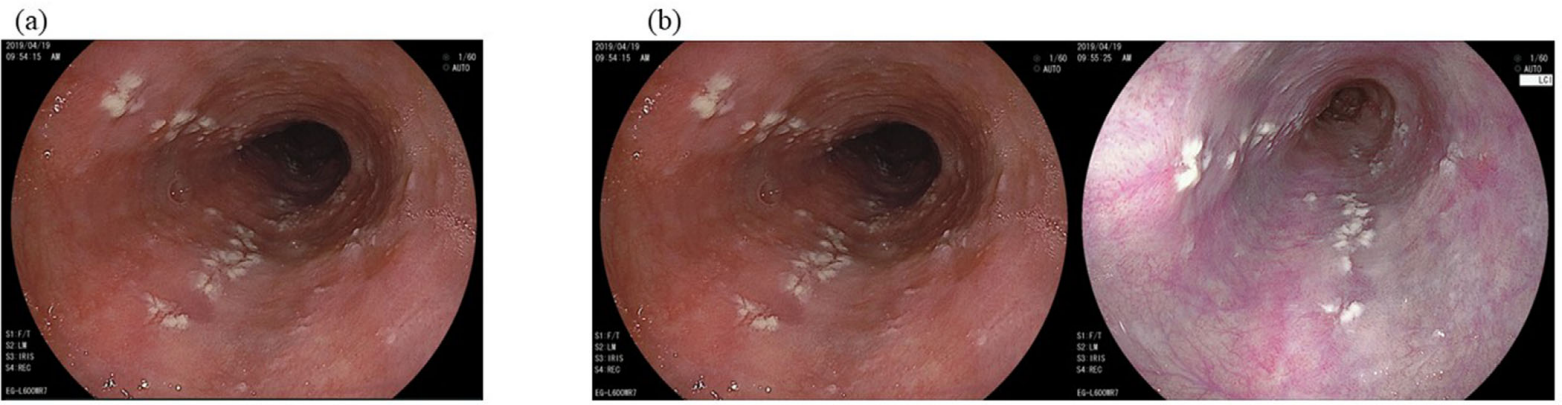
Representative images with non‐eosinophilic esophagitis (esophageal candidiasis) in this study. (a) White light image. (b) White light image (left) combined with a linked color image (right)

Seven EoE patients were on medication (on‐demand proton pump inhibitor, one; continuous proton pump inhibitor, three; topical steroid, three). All of them had persistent abnormal endoscopic findings with active inflammation of ≥15 eosinophils/high‐power field. Similar image sets consisting of WLI and WLI+LCI were arbitrarily collected from 30 patients without EoE (Table S1 and Figure 3a,b). The image sets analyzed were prepared as PowerPoint files (Microsoft Office 2019) by one of the authors (Yasuhiko Abe), who did not participate in this study as a rater of endoscopic images. All endoscopic images were recorded using high‐definition endoscopes, including EG‐L580NW, EG‐L580NW7, EG‐L590WR, EG‐L590ZW, EG‐L600WR7, and EG‐L600ZW7, with a LASEREO endoscopic system (Fujifilm, Tokyo, Japan).

### Image evaluation

Ten endoscopists independently reviewed 30 WLI and 30 WLI+LCI images in random order without any clinical information. The raters were instructed to assess each image and enter the answer into an Excel sheet and were prohibited from returning to the previous images for re‐assessment. Four of the raters had board certification from the Japan Gastroenterological Endoscopy Society (JGES; expert [Ex], mean endoscopy experience of 10.8 years), and the remaining six did not (non‐expert [non‐Ex], mean endoscopy experience of 6.8 years). Prior to the image evaluation, all raters received a lecture on the characteristic endoscopic findings of EoE for 20 min from an Ex (Yasuhiko Abe).

Five EoE‐related endoscopic findings, including edema (absent to mild or severe), ring (absent, mild, moderate, or severe), exudates (absent to mild or severe), furrows (absent or present), and stricture (absent or present) were scored according to the adjusted EoE EREFS^15^ (Table 1), a simplified version developed by Hirano et al.,^12^ with moderate to substantial inter‐ and intra‐observer agreement except for edema. The raters answered either 0, non‐EoE or 1, EoE. The entered ratings were tallied. After 8 weeks, all raters independently reviewed the 30 WLI and 30 WLI+LCI images in a different random order and re‐rated them on the same index to assess intra‐observer agreement.

**TABLE 1:** The adjusted eosinophilic esophagitis endoscopic reference score scoring system

	**0**	**1**	**2**	**3**
Edema	Absent to mild (loss of clarity of vascular marking)	Severe (absence of vascular marking)		
Rings	Absent	Mild (subtle circumferential rings)	Moderate (distinct rings, still passage of diagnostic endoscope)	severe (distinct rings, no passage of diagnostic endoscope)
Exudates	Absent to mild (<10% of esophageal surface area)	Severe (>10% of esophageal surface area)		
Furrows	Absent	Present		
Stricture	Absent	Present		

Abbreviation: EREFS, EoE endoscopic reference score.

### Diagnostic ability by WLI and WLI+LCI/inter‐ and intra‐observer agreement

The sensitivity, specificity, positive predictive value, negative predictive value, accuracy, and area under the curve (AUC) for the diagnosis of EoE were calculated and compared between WLI and WLI+LCI. The interobserver agreement between all pairs of raters for each of the five endoscopic findings and diagnosis of EoE was calculated according to Cohen's kappa statistics.^32^ The weighted kappa value was applied to assess the ring with a 4‐grade rating.^33^ All kappa values were summed, statistically analyzed, and compared between WLI and WLI+LCI and also separately compared between Ex versus Ex, Ex versus non‐Ex, and non‐Ex versus non‐Ex according to the JGES board certification. The intra‐observer agreement between the first and second assessments was calculated and summed for all nine raters and analyzed separately for Ex and non‐Ex groups in the same manner.

### Statistical analysis

The details of the statistical analysis are shown in Supplementary Methods. The kappa values were considered as follows: ≤0.20, poor; 0.21–0.4, fair; 0.41–0.60, moderate; 0.61–0.80, substantial; and 0.81–1.00, almost perfect agreement.^34^

## RESULTS

### Diagnostic ability by WLI and WLI+LCI

The diagnostic accuracy of WLI and WLI+LCI was analyzed and compared according to the presence or absence of the JGES board certification, the number of EoE patients experienced, EREFS score in EoE, and the endoscopic phenotype of EoE (Table 2). The mean EREFS score was 2.3±1.1 (standard deviation). Severe stricture with failure to pass a diagnostic endoscope was not included. Overall, WLI+LCI achieved a higher diagnostic accuracy for EoE than WLI, which was more remarkable in the non‐Exs (AUC, WLI, 0.78; WLI+LCI, 0.85), in the raters with no EoE patients experienced (AUC, WLI, 0.72; WLI+LCI, 0.82), for images with EREFS scores ≤1 (AUC, WLI, 0.61; WLI+LCI, 0.70), and in the diffuse type of EoE (AUC, WLI, 0.81; WLI+LCI, 0.89).

**TABLE 2:** Diagnistic ability of the endoscopic findings and the diagnosis of EoE

Mode	Sen	Spe	PPV	NPV	Acc	AUC
total (30 EoE and 30 non‐EoE images, 10 endoscopists)						
WLI	0.70	0.87	0.84	0.74	0.78	0.78
95% CI	0.62‐0.79	0.81‐0.93	0.77‐0.92	0.69‐0.81	0.73‐0.83	0.73‐0.83
WLI+LCI	0.79	0.90	0.89	0.81	0.85	0.85
95% CI	0.73‐0.89	0.83‐0.94	0.85‐0.95	0.78‐0.86	0.82‐0.87	0.82‐0.87
expert (30 EoE and 30 non‐EoE images, 4 endoscopists)						
WLI	0.72	0.88	0.85	0.76	0.80	0.80
95%CI	0.62‐0.82	0.75‐0.99	0.74‐0.97	0.70‐0.81	0.75‐0.84	0.75‐0.84
WLI+LCI	0.82	0.88	0.88	0.83	0.85	0.85
95%CI	0.75‐0.89	0.78‐0.99	0.76‐0.98	0.78‐0.87	0.81‐0.89	0.81‐0.89
non‐expert (30 EoE and 30 non‐EoE images, 6 endoscopists)						
WLI	0.69	0.86	0.83	0.73	0.78	0.78
95%CI	0.47‐0.86	0.76‐0.96	0.72‐0.96	0.64‐0.85	0.68‐0.87	0.68‐0.87
WLI+LCI	0.78	0.92	0.90	0.80	0.85	0.85
95%CI	0.66‐0.90	0.83‐1.00	0.83‐0.99	0.74‐0.89	0.80‐0.90	0.80‐0.90
number of EoE patient experienced ≥5 (30 EoE and 30 non‐EoE images, 4 endoscopists)						
WLI	0.74	0.87	0.85	0.77	0.80	0.80
95%CI	0.54‐0.95	0.39‐1.11	0.75‐0.95	0.67‐0.86	0.71‐0.89	0.71‐0.90
WLI+LCI	0.82	0.90	0.88	0.83	0.85	0.85
95%CI	0.79‐0.84	0.75‐1.04	0.76‐1.02	0.83‐0.83	0.80‐0.90	0.80‐0.90
number of EoE patients EoE experienced≤4 (30 EoE and 30 non‐EoE images, 3 endoscopists)						
WLI	0.76	0.88	0.86	0.78	0.82	0.82
95%CI	0.56‐0.96	0.75‐1.00	0.66‐1.01	0.67‐0.90	0.78‐0.85	0.79‐0.85
WLI+LCI	0.87	0.87	0.87	0.87	0.87	0.87
95%CI	0.71‐1.03	0.72‐1.01	0.73‐1.00	0.72‐1.02	0.73‐1.01	0.73‐1.01
no patient EoE experienced (30 EoE and 30 non‐EoE images, 3 endoscopists)						
WLI	0.59	0.86	0.80	0.68	0.72	0.72
95%CI	0.41‐0.77	0.48‐1.23	0.38‐1.27	0.51‐0.83	0.50‐0.95	0.50‐0.95
WLI+LCI	0.69	0.96	0.94	0.75	0.82	0.82
95%CI	0.48‐0.90	0.83‐1.08	0.79‐1.09	0.65‐0.85	0.78‐0.86	0.78‐0.86
EREFS score≤1 (8 EoE and 30 non‐EoE images, 10 endoscopists)						
WLI	0.35	0.87	0.41	0.83	0.76	0.61
95%CI	0.32‐0.74	0.54‐0.96	0.33‐0.65	0.82‐0.91	0.58‐0.84	0.57‐0.71
WLI+LCI	0.50	0.90	0.58	0.87	0.82	0.70
95%CI	0.37‐0.64	0.85‐0.96	0.51‐0.77	0.84‐0.90	0.79‐0.85	0.65‐0.76
EREFS score≥2 (22 EoE and 30 non‐EoE images, 10 endoscopists)						
WLI	0.83	0.87	0.82	0.87	0.85	0.85
95%CI	0.77‐0.88	0.81‐0.93	0.76‐0.90	0.84‐0.91	0.81‐0.89	0.81‐0.89
WLI+LCI	0.90	0.90	0.87	0.92	0.90	0.90
95%CI	0.84‐0.95	0.85‐0.96	0.82‐0.94	0.89‐0.96	0.86‐0.92	0.87‐0.93
diffuse type EoE (25 EoE and 30 non‐EoE images, 10 endoscopists)						
WLI	0.76	0.87	0.83	0.81	0.82	0.81
95%CI	0.68‐0.84	0.81‐0.93	0.76‐0.91	0.76‐0.87	0.77‐0.87	0.76‐0.86
WLI+LCI	0.87	0.90	0.88	0.89	0.89	0.89
95%CI	0.82‐0.93	0.85–0.96	0.84‐0.95	0.86‐0.94	0.86‐0.92	0.86‐0.92
localized type EoE (5 EoE and 30 non‐EoE images, 10 endoscopists)						
WLI	0.40	0.87	0.33	0.90	0.80	0.63
95%CI	0.31‐0.61	0.69‐0.97	0.25‐0.57	0.89‐0.92	0.67‐0.88	0.59‐0.70
WLI+LCI	0.40	0.90	0.41	0.90	0.83	0.65
95%CI	0.21‐0.59	0.85‐0.96	0.25‐0.54	0.88‐0.93	0.79‐0.87	0.57‐0.74

Sen, sensitivity; Spe, specificity; PPV, positive predictive value; NPV, negative predictive value; Acc, accuracy; AUC, area under the curve

EoE, eosinophilic esophagitis;WLI, white light image; LCI, linked color image; EREFS, EoE endoscopic reference score; CI, confidence interval

expert, endoscopists with board certification of the Japan Gastroenterological Endoscopy Society (JGES) ; non‐expert, endoscopists without board certification of the JGES

### Inter‐observer agreement

**FIGURE 4:**
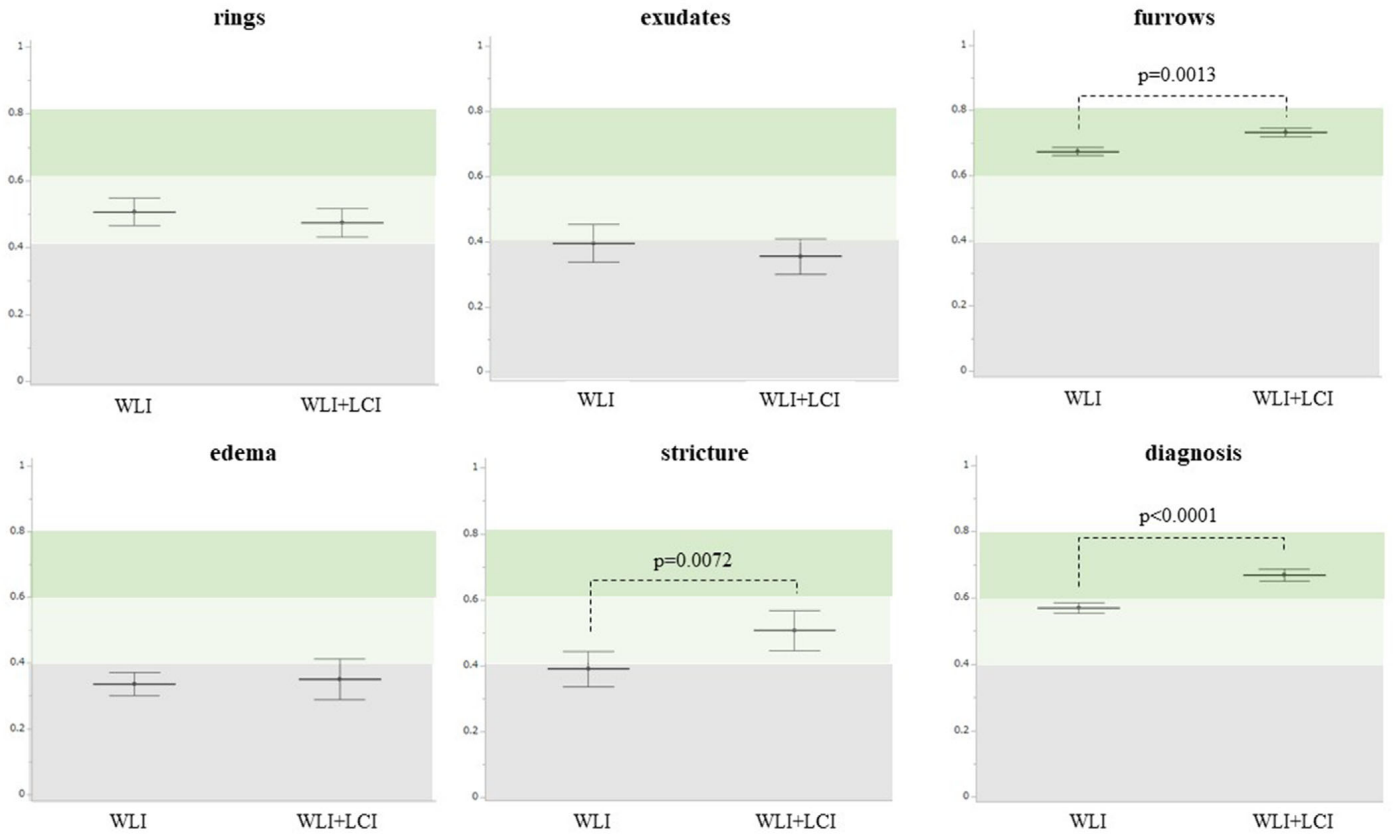
Inter‐observer agreement with WLI versus WLI+LCI. The kappa value in the WLI+LCI group was significantly higher than that in the WLI group for furrows, stricture, and diagnosis. No additional increase in kappa value by adding LCI to WLI was found in the rings, exudates, or edema. The middle horizontal lines and vertical lines represent the mean kappa value and 95% confidence interval, respectively. WLI, white light image; LCI, linked color image

**FIGURE 5:**
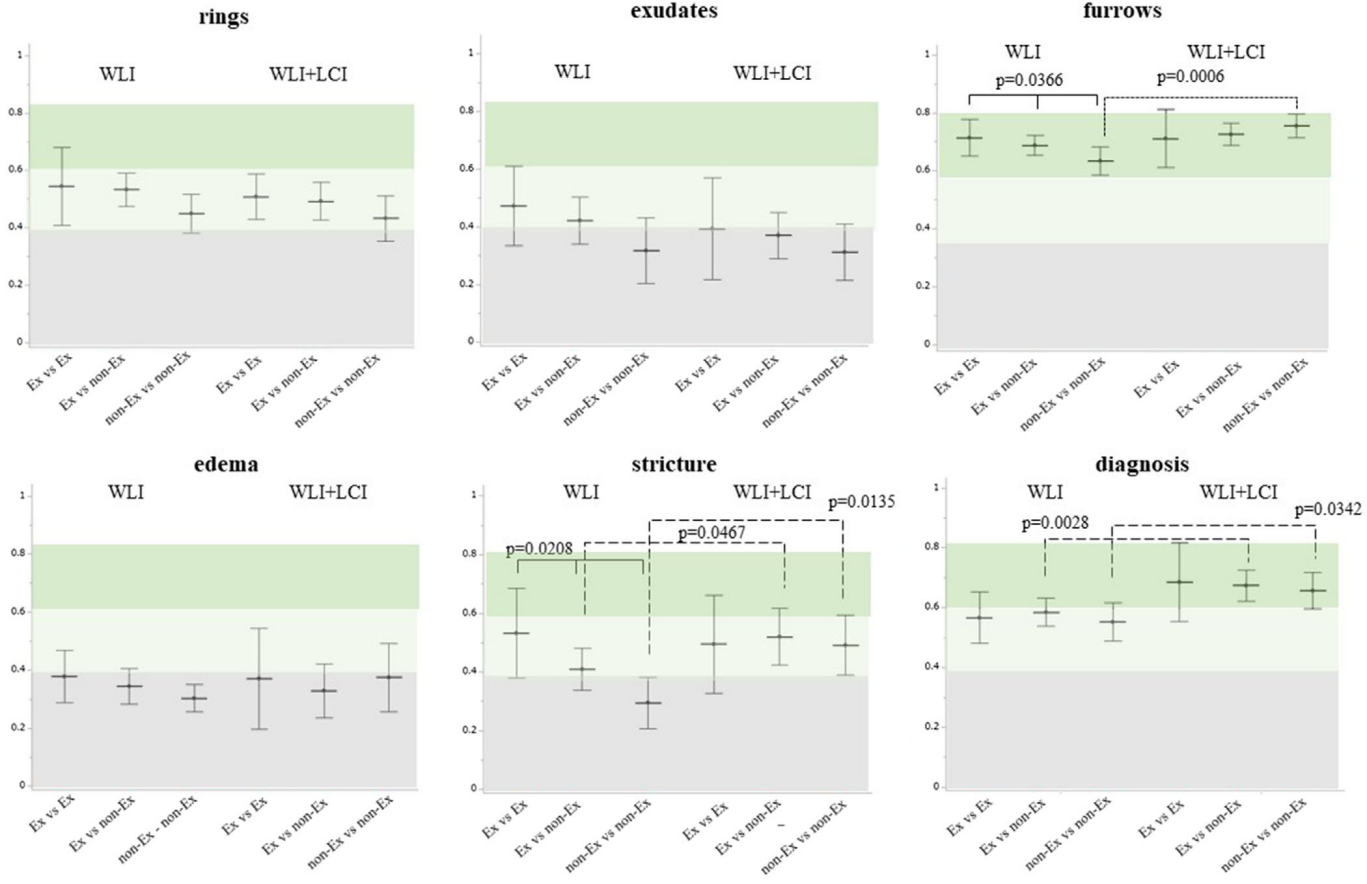
Inter‐observer agreement according to the presence or the absence of board certification of JGES. In WLI, the kappa value tended to be higher in the Ex versus Ex than in the Ex versus non‐Ex and non‐Ex versus non‐Ex group, with a statistically significant difference, especially in furrows and stricture. In WLI+LCI, the kappa value was similar in all five findings and the diagnosis among the three groups. Notably, in the non‐Ex versus non‐Ex, the kappa value in WLI+LCI was significantly higher than WLI in furrows, strictures, and diagnosis. In the Ex versus non‐Ex, a significant difference was noted between WLI and WLI+LCI in stricture and diagnosis. The middle horizontal lines and vertical lines represent the mean kappa value and 95% confidence interval, respectively. A *p*‐value was calculated by Wilcoxon ranked sign test. WLI, white light image; LCI, linked color image; JGES, the Japan Gastroenterological Endoscopy Society; Ex, the endoscopist with board certification of JGES; non‐Ex, the endoscopist without board certification of JGES

The kappa value for WLI+LCI was significantly higher than that for WLI in furrows (WLI, 0.67; WLI+LCI, 0.73, *p* = 0.0013), stricture (WLI, 0.39; WLI+LCI, 0.51, *p* = 0.0072), and diagnosis (WLI, 0.57; WLI+LCI, 0.67, *p* < 0.0001; Figure 4). No additional increase for WLI+LCI was found in the rings, exudates, or edema (Figure 4). When examined according to the presence or absence of board certification of JGES, the kappa value in WLI was higher in the Ex versus Ex group than in the Ex versus non‐Ex group and the non‐Ex versus non‐Ex group, with a statistically significant difference in furrows (Ex vs. Ex, WLI, 0.71; Ex vs. non‐Ex, 0.69; non‐Ex vs. non‐Ex, 0.63, *p* = 0.0366) and stricture (Ex vs. Ex, WLI, 0.53; Ex vs. non‐Ex, 0.41; non‐Ex vs. non‐Ex, 0.30, *p* = 0.0208) (Figure 5). In contrast, the kappa value for WLI+LCI was similar in all findings and diagnoses among the three groups (Figure 5). Notably, in the non‐Ex versus non‐Ex group, the kappa value for WLI+LCI was significantly higher than that for WLI in furrows (WLI, 0.63; WLI+LCI, 0.75, *p* = 0.0006), strictures (WLI, 0.30; WLI+LCI, 0.49, *p* = 0.0135), and diagnosis (WLI, 0.55; WLI+LCI, 0.66, *p* = 0.0342). In the Ex versus non‐Ex group, there was a statistically significant difference between WLI and WLI+LCI regarding strictures (WLI, 0.41; WLI+LCI, 0.52, *p* = 0.0467) and diagnosis (WLI, 0.52; WLI+LCI, 0.63, *p* = 0.0028) (Figure 5).

When inter‐observer agreement in diffuse EoE was sub‐analyzed, the kappa value for WLI+LCI was significantly higher than that for WLI in furrows (WLI, 0.68; WLI+LCI, 0.74, *p* = 0.0037), stricture (WLI, 0.31; WLI+LCI, 0.50, *p* = 0.0002), and diagnosis (WLI, 0.60; WLI+LCI, 0.70, *p* < 0.0001; Table S2). In the localized type of EoE, however, no additional increase with WLI+LCI was found except for edema with a considerably lower kappa value for WLI+LCI than for WLI (WLI, 0.23; WLI+LCI, 0.08, *p* = 0.0002; Table S3).

### Intra‐observer agreement

**FIGURE 6:**
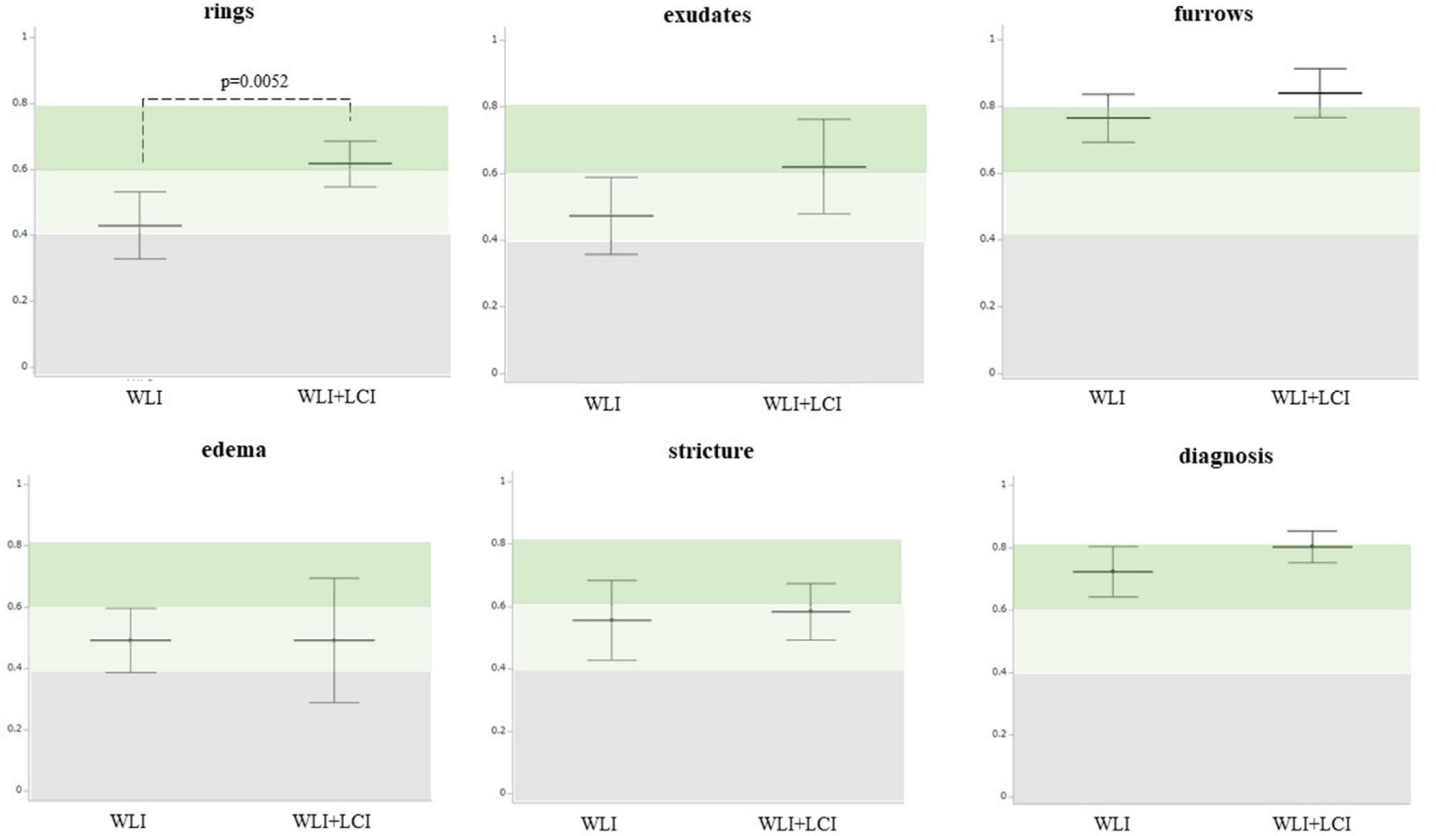
Intra‐observer agreement with WLI versus WLI+LCI. The kappa value in the ring in the WLI+LCI group was significantly higher than that in the WLI group, and this trend was found for exudates, furrows, and diagnosis. The middle horizontal and vertical lines represent the mean kappa value and 95% confidence interval, respectively. WLI, white light image; LCI, linked color image

**FIGURE 7:**
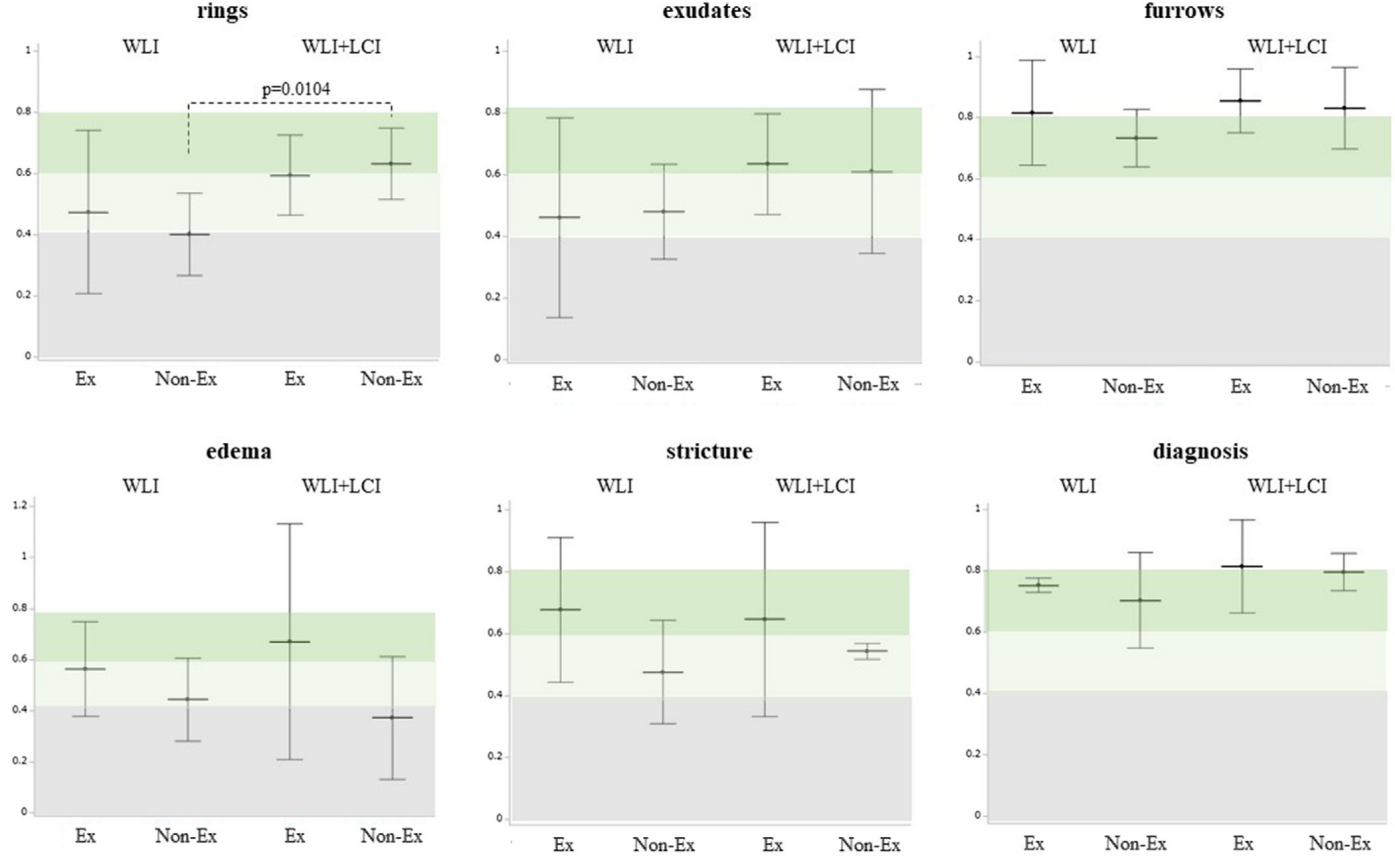
Intra‐observer agreement according to the presence or the absence of board certification of JGES. The statistically significant increase of the kappa value in WLI+LCI in rings was found in the non‐Ex, but not in the Ex. Excluding edema and stricture, the kappa values in WLI+LCI were almost equally high regardless of Ex and non‐Ex, which was slightly increased compared with those in WLI. The middle horizontal lines and the vertical lines represent the mean kappa value and 95% confidence interval, respectively. A *p*‐value was calculated by Wilcoxon ranked sign test. WLI, white light image; LCI, linked color image; JGES, the Japan Gastroenterological Endoscopy Society; Ex, the endoscopists with board certification of JGES; non‐Ex, the endoscopists without board certification of JGES

Regarding the intra‐observer agreement between WLI versus WLI+LCI, the kappa value for WLI+LCI was significantly higher than that for WLI in the ring (WLI, 0.43; WLI+LCI, 0.62, *p* = 0.0052), and this trend was found in exudates, furrows, and diagnosis (Figure 6). When examined according to the presence or absence of JGES board certification, the increased kappa value for WLI+LCI in the ring was found to be statistically significant in the non‐Ex group (WLI, 0.40; WLI+LCI, 0.63, *p* = 0.0104; Figure 7). Excluding edema and stricture, the kappa value for WLI+LCI was almost equally high, regardless of Ex and non‐Ex (substantial to almost perfect), which was slightly higher than that for WLI (Figure 7).

When intra‐observer agreement in diffuse type of EoE was sub‐analyzed, the kappa value for WLI+LCI was significantly higher than for WLI in the ring (WLI, 0.45; WLI+LCI, 0.64, *p* = 0.014), and it tended to increase in exudates, furrows, and diagnosis, as in the whole analysis (Table S4). Similarly, when the intra‐observer agreement for the localized EoE was analyzed, the kappa value for WLI+LCI was higher than that for WLI in rings without significance. Conversely, the kappa value in exudates and strictures was found to be higher for WLI than in WLI+LCI (Table S5).

## DISCUSSION

This study first demonstrated that LCI was useful for the diagnosis of EoE, especially for less‐experienced endoscopists with EoE and for cases with milder endoscopic abnormalities. The results of inter‐ and intra‐observer agreements indicated that the additional effect of LCI on WLI could be mainly attributed to the improvement of diagnostic consistency in furrows, strictures, and rings especially in diffuse types of EoE. Few reports have investigated the endoscopic diagnostic consistency of EoE, including IEE. Peery et al. reported that NBI had no additional effect on WLI in the diagnosis of EoE, in a manner similar to that in the present study.^16^ van Rhijn et al. showed that the adjusted EREFS scoring system using WLI yielded “moderate” to “substantial” inter‐ and intra‐observer agreement, except for the rating of edema, being superior to the original version by Hirano et al.^15^ In a Japanese study by Izumi et al., endoscopic diagnostic consistency for EoE and relevant endoscopic findings did not reach a clinically acceptable level with “fair” to “moderate” inter‐ and intra‐observer agreement in either with or without board‐certificated endoscopists.^17^ Differences between these studies may be due to heterogeneities in the endoscopic severity of EoE, raters’ practical experience with EoE, whether or not non‐EoE images are included, and the resolution and condition of images analyzed.

Furthermore, a meta‐analysis showed that EoE was endoscopically “normal” in 20% of retrospective studies and 7% of prospective studies.^4^ This suggests that a certain number of EoE patients might be misdiagnosed as normal by omitting biopsies even for patients with dysphagia or food impaction. Additionally, low accuracy of endoscopic diagnosis and resultant diagnostic delay without effective therapeutic intervention carry a higher risk of esophageal stricture,^35^, ^36^, ^37^ impaired quality of life,^38^ decreased treatment response,^39^ repeated esophageal dilatation,^40^ and even critical mechanical injuries, such as perforation.^41^

LCI provides clear and bright images using pre‐processing technology with radiation of short‐wavelength laser light (410 nm) and post‐processing technology with the enhancement of color difference. This allows red areas to appear redder and white areas to appear whiter while maintaining a brighter field of endoscopic view compared to WLI.^20^, ^42^ The color difference between WLI and LCI has been well‐proven in previous reports evaluating the usefulness of LCI in endoscopic diagnosis for various GI diseases.^22^, ^27^, ^43^ LCI might yield detailed surface information up to a more distant area of the esophagus and improved diagnostic ability and consistency of EoE, as presented images in Figures 1 and 3). Notably, the addition of LCI improved the diagnostic consistency of furrows, strictures, and diagnosis among endoscopists, including non‐Exs, correcting the difference in diagnostic consistency between endoscopists with and without board certification seen for WLI. LCI is expected to contribute to the screening and diagnosis of EoE in clinical practice where endoscopy is not always performed by Ex endoscopists.

There are some cases in which abnormal findings are localized to a small area at the lower end of the esophagus.^31^ We found that the diagnostic ability was much lower in the localized type than in the diffuse type, and there was no diagnostic improvement with the addition of LCI (Table 1). In the localized type, the inter‐observer agreement in edema (Table S3) and intra‐observer agreement in stricture (Table S5) were significantly higher in WLI than in WLI+LCI. A lack of images with the localized type might affect the results. The advantages of LCI might not be observed in images inherently capturing closer areas for the localized type than images analyzed in the diffuse type. The degree of endoscopic abnormality in the localized type was originally less severe than that in the diffuse type (mean EREFS score, 1.2 vs. 2.5, respectively), which may further influence the results. Meanwhile, the additional effect of LCI on the diagnostic consistency of EoE was confirmed to be statistically significant even when the localized type was excluded from the analysis (Table 1 and Tables S2 and S4).

This study had several limitations. First, the sample size was small, and appropriate sample size to detect statistical significance was not calculated because of the lack of referable reports investigating the efficacy of LCI for the diagnosis of EoE. Therefore, we referred to two previous reports investigating the usefulness of LCI for the endoscopic diagnosis of minimal change esophagitis,^28^ ^,46^ in which the addition of LCI on WLI increased the detection rate by 15%–20% compared with WLI alone, increased the inter‐observer agreement from “moderate” to “almost perfect”^28^ and “moderate” to “substantial”,^44^ and increased the intra‐observer agreement from “fair/moderate” to “moderate/substantial”^28^ and “moderate” to “substantial”.^44^ Second, we did not objectively evaluate the color difference between WLI and LCI using the CIE Lab color space system.^45^ Third, it was impossible to use the images of WLI and LCI taken under the same conditions because LCI is created by pre‐processing and post‐processing technology, although we prepared paired images with as many similar compositions as possible. In a one‐to‐one comparison of WLI and LCI, even mild differences in the composition may lead to differences in image evaluation. Since this study compared the difference between WLI and WLI+LCI, but not between WLI and LCI, we believe that the impact of subtle differences between these images is limited in assessing the additional effect of LCI on WLI. Furthermore, we are aware that dynamic observation during endoscopy is useful for the diagnosis of GI diseases. Fifth, endoscopically unnoticed EoE without biopsies might have been included in the non‐EoE group. Sixth, various esophageal conditions and diseases including relatively rare entities such as Cowden disease and drug‐induced esophagitis could be analyzed in the non‐EoE group. Although we did not analyze diagnostic ability and inter‐ and intra‐observer agreements for the non‐EoE group, Ex may diagnose more accurately in the non‐EoE group than non‐Ex and consequently, the diagnostic accuracy might be improved.

In conclusion, we showed that LCI could contribute to the improvement of the endoscopic diagnosis of EoE by enhancing the visibility of abnormal findings and reducing the diagnostic disparities between Exs and non‐Exs.

## CONFLICT OF INTEREST

The authors declare no conflict of interest.

## FUNDING INFORMATION

None.

## Supporting information


**Table S1**. Esophageal conditions whose images were collected as the non‐eosinophilic esophagitis group

**Table S2**. Inter‐observer agreement (diffuse type of eosinophilic esophagitis)

**Table S3**. Inter‐observer agreement (localized type of eosinophilic esophagitis)

**Table S4**. Intra‐observer agreement (diffuse type of eosinophilic esophagitis)

**Table S5**. Intra‐observer agreement (localized type of eosinophilic esophagitis)

Supplementary Methods
