# Supplementary material for: Linked color imaging improves the diagnostic accuracy of eosinophilic esophagitis
Source: DEN Open. 2022 Jul 25;3(1):e146. doi: 10.1002/deo2.146 (PMC9310047; doi:10.1002/deo2.146)
Supplement: Supplementary file 6 — Supplementary Methods [file DEO2-3-e146-s006.docx]

**Supplementary methods**

Statistical analysis was performed using the JMP® 14.3.0 software program (SAS Institute, Cary, NC, USA) and R programming language software version 3.6.1 (https://cran.r-project.org/bin/windows/base/old/3.6.1/). The mean kappa value with 95% confidence interval (CI) was calculated for each of the WLI and WLI+LCI images. The Wilcoxon signed-rank test and Kruskal–Wallis test were used to compare kappa values between the two groups and the three groups, respectively. The adjusted EREFS scores were determined by two experienced endoscopists (Y.A. and Y. S.) using WLI images collected from EoE patients, and additional analysis was conducted based on the severity of the EREFS score. Statistical significance was set at p value < 0.05.
